# Optimizing medical care for geriatric patients in Austria: defining a top five list of “Choosing Wisely” recommendations using the Delphi technique

**DOI:** 10.1007/s41999-018-0105-8

**Published:** 2018-09-27

**Authors:** Walter Schippinger, Anna Glechner, Karl Horvath, Ulrike Sommeregger, Thomas Frühwald, Peter Dovjak, Georg Pinter, Bernhard Iglseder, Peter Mrak, Walter Müller, Gerald Ohrenberger, Eva Mann, Birgit Böhmdorfer, Regina Roller-Wirnsberger

**Affiliations:** 1 Department of Internal Medicine and Acute Geriatrics, Geriatric Health Centres Graz, Albert Schweitzer Hospital, Graz, Austria; 2 Department for Evidence-based Medicine and Clinical Epidemiology, Danube University Krems, Krems an der Donau, Austria; 3 Institute of General Practice and Evidence-Based Health Services Research, Medical University of Graz, Graz, Austria; 4 Department of Acute Geriatrics, Social Medical Center East, Vienna, Austria; 5 Department of Acute Geriatrics and Remobilisation, Hospital of Salzkammergut, Gmunden, Austria; 6 Department of Acute Geriatrics, Hospital of Klagenfurt, Klagenfurt, Austria; 7 Department of Geriatrics, Christian-Doppler University Hospital Salzburg, Private Medical University Paracelsus, Salzburg, Austria; 8 Department of Internal Medicine 2, General Hospital West-Styria, Voitsberg, Austria; 9 Department of Acute Geriatrics and Remobilisation, General Public Hospital of the Order of Saint Elisabeth, Klagenfurt, Austria; 10 House of Mercy, Long Term Care Hospital, Vienna, Austria; 11 Private Practice for General Medicine, Rankweil, Austria; 12 Institute for General, Family and Preventive Medicine, Private Medical University Paracelsus, Salzburg, Austria; 13 Pharmacy Department, Hospital Hietzing with Neurological Centre Rosenhügel, Vienna, Austria; 14 Department of Internal Medicine, Medical University of Graz, Auenbruggerplatz 15, 8036 Graz, Austria

**Keywords:** Geriatric medicine, Choosing Wisely, Recommendations, Consensus, Delphi process

## Abstract

**Purpose:**

Inappropriate use of diagnostic and therapeutic medical procedures is common and potentially harmful for older patients. The Austrian Society of Geriatrics and Gerontology defined a consensus of five recommendations to avoid overuse of medical interventions and to improve care of geriatric patients.

**Methods:**

From an initial pool of 147 reliable recommendations, 20 were chosen by a structured selection process for inclusion in a Delphi process to define a list of five top recommendations for geriatric medicine. 12 experts in the field of geriatric medicine scored the recommendations in two Delphi rounds.

**Results:**

The final five recommendations are concerning urinary catheters in elderly patients, percutaneous feeding tubes in patients with advanced dementia, antipsychotics as the first choice to treat behavioral and psychological symptoms of dementia, and screening for breast, colorectal, prostate, or lung cancer, and the use of antimicrobials to treat asymptomatic bacteriuria.

**Conclusions:**

The selected recommendations have the potential to improve medical care for older patients, to reduce side effects caused by unnecessary medical procedures, and to save costs in the health care system.

## Introduction

Currently, Europe has the largest proportion of people in the age of 60 years and above in the world [1]. The oldest population group of people 80 years and older is expected to grow from 137 million in 2017 to 425 million in the year 2050 worldwide [1]. Higher age is a major risk factor for multiple morbidities and impaired functional capacities [2].

Health care systems, traditionally focussing on single disease management, have not yet fully adapted to the changing health care needs of an aging population presenting with multimorbidity and associated polypharmacy, geriatric syndromes, and reduced functional resilience [3–6]. Lack of coordination between attending physicians of different medical disciplines can result in the ineffective and inadequate treatment of multimorbid and frail persons [7]. Geriatric medicine, as a specialty of internal medicine, is not yet established in some European countries. Chronic care for older patients is in the hands of primary care physicians and other specialties not specifically trained for the distinct care needs of older patients. In several European countries, the undergraduate education and training in geriatric medicine has been reported as inadequate in various studies [8–11]. Efforts to include geriatric content in curricula for the training of medical doctors are ongoing across Europe. However, it will take years until changes in education and training will result in sustainable changes in daily practice focussed on older people.

One option to accelerate change towards better care for older patients is the introduction of practice guidelines into clinical work [12]. In 2012, the American Board of Internal Medicine Foundation (ABIMF) launched an initiative called “Choosing Wisely” questioning the impact of diagnostic and therapeutic procedures in certain clinical situations and with specific target groups. “Top five” lists of medical procedures performed too often and without supporting evidence in daily clinical practice were developed on evidence- and eminence-based criteria by medical specialty societies [13]. The ongoing Choosing Wisely initiative aims at fostering communication between patients and physicians about what is appropriate and beneficial treatment. Choosing Wisely has published about 500 recommendations regarding 75 medical specialty societies, including the American Geriatrics Society [14]. During the last few years, Choosing Wisely initiatives have been launched in several other countries [15]. It is the aim of the work presented in this publication to develop in a national Choosing Wisely initiative called “gemeinsam gut entscheiden” recommendations for the management of geriatric patients in Austria. Geriatric patients were defined according to the definition of the European Union of Medical Specialists [16].

## Methods

### Literature search

All published recommendations of the US Choosing Wisely initiative were identified through the website of the American Board of Internal Medicine Foundation [17]. Additionally, a search for recommendations from Mid-European Choosing Wisely initiatives through the websites of the DianaHealth project of the Centro de Investigación Biomédica en Red de Epidemiología y Salud Pública [18] and the Less is More project was performed [19]. The literature searches were performed in April 2017. Recommendations were judged to be trustworthy if they had equivalent recommendations in German S3-guidelines or if the development process was judged to be of high methodological quality and meta-literature supporting the recommendation was cited [20].

### Selection of experts

Raters for the consensus process were selected according to clinical and academic expertise in the field of geriatric medicine. An attempt was made to collect the broadest possible range of national expert opinion. The core group included geriatricians with academic background and working in university setting, geriatricians with focus on clinical work in acute geriatric wards and in primary care as well as one clinical pharmacist working in an acute care hospital and dealing with older patients across various specialties in hospital. All of the experts who were invited were members of the academic or executive board and of expert groups of the Austrian Society of Geriatrics and Gerontology at the time of their invitation [21]. The experts were invited by e-mail and asked for their willingness to participate in the consensus process. All of the experts signed a conflict of interest form to substantiate their neutrality in evaluating the items.

### The Delphi process

The Delphi technique is a well-established consensus-finding method that is used to determine the extent of agreement among panellists regarding a specific query [22–24]. The authors used a modified Delphi process with the aim of creating a top five list of tests and treatments that have little or no demonstrable benefit, or that can be harmful. The survey was conducted anonymously in German from November 2017 to January 2018 using the online survey platform Survey Monkey™. In the Delphi survey rounds, experts were asked to rate each item regarding its clinical relevance using a 5-point Likert scale from 1 (less important) to 5 (very important). For each recommendation, a mean score of Likert scale assessments and standard deviations was calculated. All expert ratings from the first round of the Delphi survey were summarized and ranked by their mean score. The items were transcribed then into a new version of the template and all 20 items were sent out along with an overall result from the first round to all raters for a second evaluation. To assess the degree of consistency among the experts’ scores, an intra-class correlation coefficient based on a two-way random effect model using IBM SPSS (International Business Machines Corporation—Statistical Package for the Social Sciences) was calculated.

## Results

### Selection of medical recommendations for the Delphi process

**Fig. 1:**
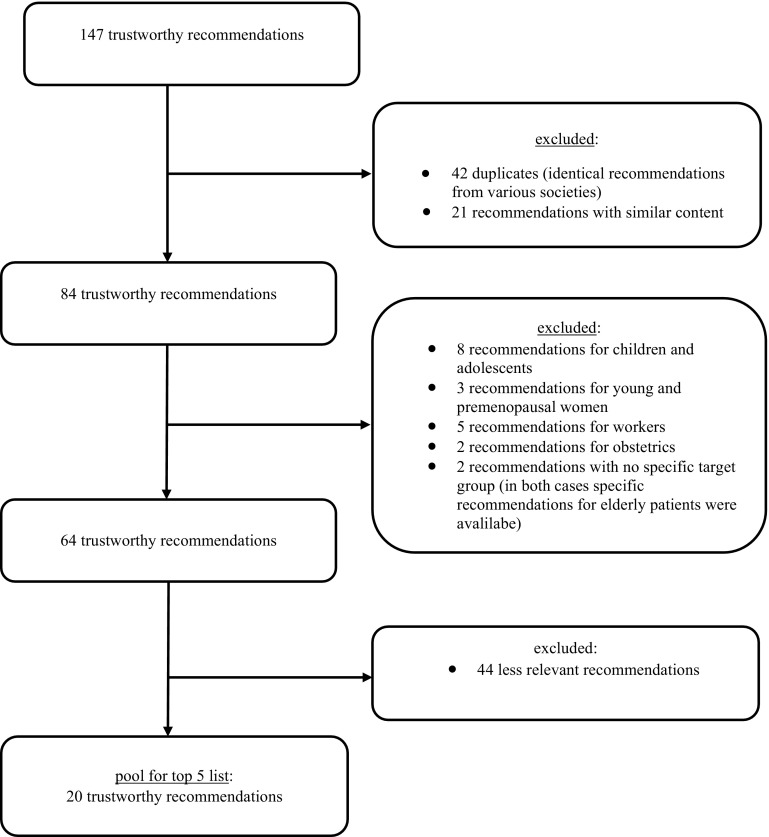
Preselection of trustworthy recommendations for inclusion in the Delphi process

Figure 1 displays the process for selecting Choosing Wisely recommendations from those published in the scientific literature, and on authorized web-based platforms and homepages. 147 recommendations were identified by our initial literature search. We excluded 42 duplicates (identical recommendations from various medical specialist societies) and 21 recommendations with similar content. Another 18 recommendations were excluded as they were not relevant for older people: recommendations for children and adolescents (8 items), young women and pre-menopausal women (3 items), occupational medicine (5 items), and obstetrics (2 items). In addition, two recommendations without a specific target group were excluded, as in both cases, a special recommendation for older, geriatric people already existed. From the remaining pool of 64 items, a core study group selected the most relevant recommendations for avoiding unnecessary tests and therapy in daily clinical practice that should be considered for further evaluation. Finally, 20 trustworthy recommendations were available for the top five lists and for assessment by experts (Fig. 1). The 20 recommendations used in the primary template for the Delphi process are shown in Table 1.

**Table 1:** 20 recommendations included in the Delphi process to define the top five list of the most important recommendations for improvement of geriatric medicine practice

Recommendation	Medical society	References
Do not obtain a Clostridium difficile toxin test to confirm “cure” if symptoms have resolved	AMDA—The Society for Post-Acute and Long-Term Care Medicine	American Board of Internal Medicine (ABIM) Foundation. Choosing Wisely. 2017. http://www.choosingwisely.org/clinician-lists/amda-c-difficile-toxin-test/. Accessed 10 Apr 2017
Do not initiate antihypertensive treatment in individuals ≥ 60 years of age for systolic blood pressure < 150 mmHg or diastolic blood pressure < 90 mmHg	AMDA—The Society for Post-Acute and Long-Term Care Medicine	American Board of Internal Medicine (ABIM) Foundation. Choosing Wisely. 2017. http://www.choosingwisely.org/clinician-lists/amda-antihypertensive-treatment-in-individuals-sixty-and-over/. Accessed 10 Apr 2017
Do not use sliding scale insulin for long-term diabetes management for individuals residing in the nursing home	AMDA—The Society for Post-Acute and Long-Term Care Medicine	American Board of Internal Medicine (ABIM) Foundation. Choosing Wisely. 2017. http://www.choosingwisely.org/clinician-lists/amda-sliding-scale-insulin-for-long-term-diabetes-management/. Accessed 10 Apr 2017
Do not insert percutaneous feeding tubes in individuals with advanced dementia. Instead, offer oral assisted feedings	AMDA—The Society for Post-Acute and Long-Term Care Medicine	American Board of Internal Medicine (ABIM) Foundation. Choosing Wisely. 2017. http://www.choosingwisely.org/clinician-lists/amda-percutaneous-feeding-tubes-for-patients-with-dementia/. Accessed 10 Apr 2017
Do not prescribe antipsychotic medications as first choice to treat behavioral and psychological symptoms of dementia and without an assessment for an underlying cause of the behavior	American Geriatrics Society, Society of General Internal Medicine AMDA—The Society for Post-Acute and Long-Term Care Medicine	American Board of Internal Medicine (ABIM) Foundation. Choosing Wisely. 2017. http://www.choosingwisely.org/clinician-lists/american-geriatrics-society-antipsychotics-for-dementia/. Accessed 10 Apr 2017 American Board of Internal Medicine (ABIM) Foundation. Choosing Wisely. 2017. http://www.choosingwisely.org/clinician-lists/antipsychotic-medications-for-dementia/. Accessed 10 Apr 2017
Do not recommend screening for breast, colorectal, prostate or lung cancer without considering life expectancy and the risks of testing, overdiagnosis and overtreatment	American Geriatrics Society, Society of General Internal Medicine AMDA—The Society for Post-Acute and Long-Term Care Medicine Society of General Internal Medicine	American Board of Internal Medicine (ABIM) Foundation. Choosing Wisely. 2017. http://www.choosingwisely.org/clinician-lists/american-geriatrics-society-breast-colorectal-prostate-cancer-screening-in-older-adults/. Accessed 10 Apr 2017 American Board of Internal Medicine (ABIM) Foundation. Choosing Wisely. 2017. http://www.choosingwisely.org/clinician-lists/amda-cancer-screenings-if-life-expectancy-less-than-10-years/. Accessed 10 Apr 2017 American Board of Internal Medicine (ABIM) Foundation. Choosing Wisely. 2017. http://www.choosingwisely.org/clinician-lists/society-general-internal-medicine-cancer-screening-in-adults-with-life-expectancy-less-than-10-years/. Accessed 10 Apr 2017
Do not use antimicrobials to treat bacteriuria in older adults unless specific urinary tract symptoms are present	American Geriatrics Society	American Board of Internal Medicine (ABIM) Foundation. Choosing Wisely. 2017. http://www.choosingwisely.org/clinician-lists/american-geriatrics-society-antimicrobials-to-treat-bacteriuria-in-older-adults/. Accessed 10 Apr 2017
Do not place, or leave in place, urinary catheters for incontinence or convenience or monitoring of output for non-critically ill patients (acceptable indications: critical illness, obstruction, hospice, preoperatively for < 2 days for urologic procedures; use weights instead to monitor diuresis)	Society of Hospital Medicine—Adult Hospital Medicine AMDA—The Society for Post-Acute and Long-Term Care Medicine; American College of Emergency Physicians American Academy of Nursing	American Board of Internal Medicine (ABIM) Foundation. Choosing Wisely. 2017. http://www.choosingwisely.org/clinician-lists/society-general-internal-medicine-peripherally-inserted-central-catheters-for-patient-provider-convenience/. Accessed 10 Apr 2017 American Board of Internal Medicine (ABIM) Foundation. Choosing Wisely. 2017. http://www.choosingwisely.org/clinician-lists/amda-indwelling-urinary-catheter/. Accessed 10 Apr 2017 American Board of Internal Medicine (ABIM) Foundation. Choosing Wisely. 2017. http://www.choosingwisely.org/clinician-lists/american-college-emergency-physicians-indwelling-urinary-catheters-in-the-emergency-department/. Accessed 10 Apr 2017 American Board of Internal Medicine (ABIM) Foundation. Choosing Wisely. 2017. http://www.choosingwisely.org/clinician-lists/american-academy-nursing-urinary-catheters-without-specific-indication/. Accessed 10 Apr 2017
Avoid ordering a brain computed tomography or brain magnetic resonance imaging to evaluate an acute concussion unless there are progressive neurological symptoms, focal neurological findings on exam or there is concern for a skull fracture	American Medical Society for Sports Medicine	American Board of Internal Medicine (ABIM) Foundation. Choosing Wisely. 2017. http://www.choosingwisely.org/clinician-lists/american-medical-society-sports-medicine-brain-ct-or-mri-to-evaluate-acute-concussion/. Accessed 10 Apr 2017
Do not perform screening for cervical cancer in low-risk women aged 65 years or older and in women who have had a total hysterectomy for benign disease	American College of Preventive Medicine American Academy of Family Physicians	American Board of Internal Medicine (ABIM) Foundation. Choosing Wisely. 2017. http://www.choosingwisely.org/clinician-lists/american-college-preventive-medicine-cervical-cancer-screening/. Accessed 10 Apr 2017 American Board of Internal Medicine (ABIM) Foundation. Choosing Wisely. 2017. http://www.choosingwisely.org/clinician-lists/american-academy-family-physicians-cervical-cancer-screening/. Accessed 10 Apr 2017
Do not screen for carotid artery stenosis in asymptomatic adult patients	American Academy of Family Physicians	American Board of Internal Medicine (ABIM) Foundation. Choosing Wisely. 2017. http://www.choosingwisely.org/clinician-lists/american-academy-family-physicians-carotid-artery-stenosis/. Accessed 10 Apr 2017
Do not perform imaging of the carotid arteries for simple syncope without other neurologic symptoms	American Academy of Neurology	American Board of Internal Medicine (ABIM) Foundation. Choosing Wisely. 2017. http://www.choosingwisely.org/clinician-lists/american-academy-neurology-carotid-artery-imaging-for-simple-syncope/. Accessed 10 Apr 2017
Avoid computed tomography pulmonary angiography in emergency department patients with a low-pretest probability of pulmonary embolism and either a negative Pulmonary Embolism Rule-Out-Criteria (PERC) or a negative d-dimer	American College of Emergency Physicians American College of Chest Physicians and American Thoracic Society	American Board of Internal Medicine (ABIM) Foundation. Choosing Wisely. 2017. http://www.choosingwisely.org/clinician-lists/acep-ct-pulmonary-angiography-in-ed-patients/. Accessed 10 Apr 2017 American Board of Internal Medicine (ABIM) Foundation. Choosing Wisely. 2017. http://www.choosingwisely.org/clinician-lists/ american-college-of-chest-physicians-american-thoracic-society-chest-ct-angiography-to-evaluate-possibly-pulmonary-embolism/. Accessed 10 Apr 2017
Avoid computed tomography scans of the head in emergency department patients with minor head injury who are at low risk based on validated decision rules	American College of Emergency Physicians	American Board of Internal Medicine (ABIM) Foundation. Choosing Wisely. 2017. http://www.choosingwisely.org/clinician-lists/american-college-emergency-physicians-ct-scans-of-head-for-emergency-department-patients-with-minor-head-injury/. Accessed 10 Apr 2017
Do not routinely repeat dual energy X-ray absorptiometry (DXA) scans more often than once every 2 years	American College of Rheumatology	American Board of Internal Medicine (ABIM) Foundation. Choosing Wisely. 2017. http://www.choosingwisely.org/clinician-lists/american-college-rheumatology-routine-repeat-dxa-scans-more-than-once-every-two-years/. Accessed 10 Apr 2017
Don’t order apolipoprotein E genetic testing as a predictive test for Alzheimer disease	The American College of Medical Genetics and Genomics	American Board of Internal Medicine (ABIM) Foundation. Choosing Wisely. 2017. http://www.choosingwisely.org/clinician-lists/american-college-medical-genetics-genomics-apoe-genetic-testing-to-predict-alzheimer-disease/. Accessed 10 Apr 2017
Avoid colorectal cancer screening tests on asymptomatic patients with a life expectancy of less than 10 years and no family or personal history of colorectal neoplasia	American College of Surgeons	American Board of Internal Medicine (ABIM) Foundation. Choosing Wisely. 2017. http://www.choosingwisely.org/clinician-lists/american-college-surgeons-colorectal-cancer-screening-tests/. Accessed 10 Apr 2017
Do not do imaging of the spine in patients with non-specific acute low back pain and without red flags	American Academy of Physical Medicine and Rehabilitation American Academy of Family Physicians North American Spine Society American College of Physicians American Association of Neurological Surgeons and Congress of Neurological Surgeons American Society of Anesthesiologists—Pain Medicine	American Board of Internal Medicine (ABIM) Foundation. Choosing Wisely. 2017. http://www.choosingwisely.org/clinician-lists/aapmr-imaging-for-back-pain/. Accessed 10 Apr 2017 American Board of Internal Medicine (ABIM) Foundation. Choosing Wisely. 2017. http://www.choosingwisely.org/clinician-lists/american-academy-family-physicians-imaging-low-back-pain/. Accessed 10 Apr 2017 American Board of Internal Medicine (ABIM) Foundation. Choosing Wisely. 2017. http://www.choosingwisely.org/clinician-lists/north-american-spine-society-advanced-imaging-of-spine-within-first-six-weeks-of-non-specific-acute-low-back-pain/. Accessed 10 Apr 2017 American Board of Internal Medicine (ABIM) Foundation. Choosing Wisely. 2017. http://www.choosingwisely.org/clinician-lists/american-college-physicians-imaging-for-non-specific-low-back-pain/. Accessed 10 Apr 2017 American Board of Internal Medicine (ABIM) Foundation. Choosing Wisely. 2017. http://www.choosingwisely.org/clinician-lists/american-association-neurological-surgeons-imaging-for-nonspecific-acute-low-back-pain/. Accessed 10 Apr 2017 American Board of Internal Medicine (ABIM) Foundation. Choosing Wisely. 2017. http://www.choosingwisely.org/clinician-lists/american-society-anesthesiologists-imaging-studies-for-acute-low-back-pain/. Accessed 10 Apr 2017
Do not recommend bed rest for more than 48 h when treating low back pain	American Academy of Physical Medicine and Rehabilitation North American Spine Society	American Board of Internal Medicine (ABIM) Foundation. Choosing Wisely. 2017. http://www.choosingwisely.org/clinician-lists/aapmr-bed-rest-for-acute-low-back-pain/. Accessed 10 Apr 2017 American Board of Internal Medicine (ABIM) Foundation. Choosing Wisely. 2017. http://www.choosingwisely.org/clinician-lists/north-american-spine-society-bed-rest-for-more-than-48-hours/. Accessed 10 Apr 2017

### First Delphi round

From the 15 experts in geriatric medicine who were invited to participate in the consensus-finding process, 12 experts responded and took part in the whole process. From the 20 recommendations presented in the first Delphi round, the following items received the highest mean scores: the overuse of urinary catheters in older patients, the percutaneous feeding tubes in patients with advanced dementia, the use of antipsychotics as the first choice for treating the behavioral and psychological symptoms of dementia, the screening for breast, colorectal, prostate, or lung cancer in people with limited life expectancy, and the use of antimicrobials to treat asymptomatic bacteriuria. The results of the first round of the Delphi process are shown in Table 2.

**Table 2:** Results of Delphi round 1, rated by 12 assessors

Recommendation	Ratings Likert Scale^a^					Mean value	Standard deviation	Median value/interquartile range^c^	Position of item according to mean value
	1	2	3	4	5				
Do not place, or leave in place, urinary catheters for incontinence or convenience or monitoring of output for non-critically ill patients (acceptable indications: critical illness, obstruction, hospice, preoperatively for < 2 days for urologic procedures; use weights instead to monitor diuresis)	0	0	0	2	10	4.83	0.389	5.0/5.0–5.0	1
Do not recommend percutaneous feeding tubes in patients with advanced dementia; instead offer oral assisted feeding	0	0	2	1	9	4.58	0.793	5.0/4.25–5.0	2
Do not use antipsychotics as the first choice to treat behavioral and psychological symptoms of dementia	1	0	1	2	8	4.33	1.231	5.0/4.0–5.0	3
Do not recommend screening for breast, colorectal, prostate or lung cancer without considering life expectancy and the risks of testing, overdiagnosis and overtreatment	0	1	1	4	6	4.25	0.965	5.0/4.0–5.0	4
Do not use antimicrobials to treat bacteriuria in older adults unless specific urinary tract symptoms are present	0	1	2	3	6	4.17	1.030	5.0/3.25–5.0	5
Intraclass correlation coefficient^b^						0.79, 95% CI 0.59–0.92			

^a^Likert scale: 1 = less important, 5 = very important

^b^Two-way random effects model

^c^Range 25th to 75th percentile

However, there was inconsistency in the ratings by the experts during the first Delphi round. While each of the five items with the highest mean value achieved scores of 4 or 5 with at least 75% of the raters, one item was rated with a score of 1 by one panel member and two items were given a score of 2 indicating the necessity for a second consensus round. The intra-class correlation coefficient of the ratings was 0.79, 95% CI 0.59–0.92.

### Second Delphi round

All raters participating in round one also completed Delphi round two. In the second round, a consensus for the five most important recommendations could be defined (Table 3). The second Delphi round demonstrated an even broader agreement on the top five recommendations from round one with over 90 percent of the top five items from round one receiving scores of 4 or 5 (mean scores 4.5–4.8, standard deviation 0.4–0.7). The calculated intra-class correlation coefficient of the raters’ votes of Delphi round two was 0.73, 95% CI 0.52–0.89.

**Table 3:** Results of Delphi round 2, rated by 12 assessors

Recommendation	Ratings Likert Scale^a^					Mean value	Standard deviation	Median value/interquartile range^c^	Position of item according to mean value
	1	2	3	4	5				
Do not place, or leave in place, urinary catheters for incontinence or convenience or monitoring of output for non-critically ill patients (acceptable indications: critical illness, obstruction, hospice, preoperatively for < 2 days for urologic procedures; use weights instead to monitor diuresis)	0	0	0	2	10	4.83	0.389	5.0/5.0–5.0	1
Do not recommend percutaneous feeding tubes in patients with advanced dementia; instead offer oral assisted feeding	0	0	0	3	9	4.75	0.452	5.0/4.25–5.0	2
Do not use antimicrobials to treat bacteriuria in older adults unless specific urinary tract symptoms are present	0	0	1	2	9	4.67	0.651	5.0/4.25–5.0	3
Do not use antipsychotics as the first choice to treat behavioral and psychological symptoms of dementia	0	0	0	5	7	4.58	0.515	5.0/4.0–5.0	4
Do not recommend screening for breast, colorectal, prostate or lung cancer without considering life expectancy and the risks of testing, overdiagnosis and overtreatment	0	0	1	4	7	4.50	0.674	5.0/4.0–5.0	5
Intraclass correlation coefficient^b^						0.73, 95% CI 0.52–0.89			

^a^Likert scale: 1 = less important, 5 = very important

^b^Two-way random effects model

^c^Range 25th to 75th percentile

## Discussion

Evaluations of care pathways for older patients in Austria recorded potentially harmful diagnostic and therapeutic procedures in geriatric patients without expected benefit or medical indication [25]. Therefore, national care providers and the Austrian Society of Geriatrics and Gerontology in collaboration with two public universities in Austria initiated the development of Choosing Wisely recommendations for the management of geriatric patients in Austria. These recommendations have become necessary in medicine as rapid advances in diagnostic and therapeutic options have increased not only the appropriate use of those options, but also their unnecessary and sometimes harmful use [26]. The Choosing Wisely initiative aims at promoting greater patient involvement in the decision-making and treatment planning process. In other countries also, several similar campaigns have started with the same purpose [27–29]. However, Choosing Wisely top five lists may be confronted with criticism for lacking strict methodological requirements in the process of their development [30]. This uncertainty about evidence has an impact on their acceptance and application by physicians, and uncertain evidence of recommendations can possibly lead to harm for patients. For this reason, various expert groups have developed top five lists on the basis of solid scientific evidence and well-defined selection processes [31, 32]. For the development of the Austrian top five lists for geriatric medicine as presented here, only recommendations that fulfilled the methodological quality criteria of German S3 guidelines and guidelines based on a methodologically well-performed development process were included [20]. This strengthens the trustworthiness and the safety of these recommendations.

Compared to recommendations already existing to avoid medical overuse in the management of geriatric patients from the US [33], Canada [34], and Australia [35], the Austrian recommendations are in line with the major international topics in geriatric care. Interestingly, Austrian geriatricians see a strong demand to avoid overuse of urinary catheter placements in geriatric patients. Recently, Rossi and colleagues could show in an Italian cohort of 427 older in-hospital patients that the placement of urinary catheters was a predictor of intercurrent clinical events, such as delirium and infections, and therefore, prolonged hospital stays and worsened clinical outcomes [36]. The ESAMED study group found similar data, demonstrating an additional functional decline following hospital stays in patients unnecessarily treated with urinary catheters [37].

Recommendation number five from the Austrian “gemeinsam gut entscheiden” list for geriatric patients differs from all of the other published recommendations and addresses the overuse of diagnostic procedures to detect malignancies in the elderly population on a routine basis. There is currently no evidence in the literature that cancer screening programs are effective and efficient in the care of geriatric patients. In Austria, the national health care system provides access to such diagnostic procedures up to an advanced age without cost to the patient. The panel members of the “gemeinsam gut entscheiden” committee found this issue to be so significant and common in the care of older people in Austria that they included this recommendation in the top five lists. Obviously, due to different health care systems, this recommendation is not listed as a priority in other lists of Choosing Wisely initiatives for geriatric patients. This fact underlines the importance of developing national recommendations for certain patient groups. Patients with complex care needs are major consumers in health care systems and account for a high percentage of the costs in the health systems. Integration of care pathways for those clients has become a priority for many health care systems [38]. A shared guide for standards of practice will be useful in the treatment of geriatric patients when “traditional” guidelines fail to address their complex needs. This is the first step towards a common effort to drive health care systems towards integrated care at least for geriatric patients [39]. Especially in those countries where geriatric medicine is not yet established as a medical specialty, recommendations of Choosing Wisely initiatives may help to raise awareness regarding the complexity of care for geriatric patients in daily clinical work.

Finally, it will be important to inform a broad public audience and to enhance older people’s and patients’ acceptance of the recommendations. As shown in the literature, physicians’ perceptions of the unacceptability for patients of applying Choosing Wisely recommendations appear to be a major barrier towards implementation [40]. Geriatricians will have to share their professional expertise with other physicians to modify their practice styles and to inform patients in a shared decision-making process to support patients and to avoid unnecessary and possibly harmful medical procedures. It may also be argued that the lack of indicators measuring the impact of recommendations on quality of care is a major drawback of the work presented, but there are already data in the literature that addresses this issue. Colleagues from the Harvard Medical School, Department of Health Care Policy, have tried to address this challenge creating 26 indicators, clustered in 6 categories, to determine low value services for patients in the surgical care setting using Choosing Wisely criteria. Implementing those indicators and aligning outcomes with costs, they found that the recommendations affected only a modest percentage of the expenses, while affecting a substantial proportion of care beneficiaries [41]. So far, comparable data for the effects of Choosing Wisely recommendations in the geriatric care management are missing in literature.

It can be concluded that the recommendations from the list of the Austrian Choosing Wisely initiative “gemeinsam gut entscheiden” have the potential to improve medical care for older patients and to reduce side effects caused by unnecessary medical procedures. In addition, the application of these guidelines can save costs in the health care system, which has not been evaluated in studies up until now. Future studies should focus on the economic effects of Choosing Wisely initiatives.

## References

[1] United Nations (2017). World population prospects. The 2017 revision.

[2] Boyd CM, Fortin M (2010). Future of multimorbidity research: how should understanding of multimorbidity inform health system design?. Public Health Rev.

[3] Marengoni A, Angleman S, Melis R, Mangialasche F, Karp A, Garmen A, Meinow B, Fratiglioni F (2011). Aging with multimorbidity: a systematic review of the literature. Ageing Res Rev.

[4] American Geriatrics Society Expert Panel on the Care of Older Adults with Multimorbidity (2012). Guiding principles for the care of older adults with multimorbidity: an approach for clinicians. J Am Geriatr Soc.

[5] American Geriatrics Society Expert Panel on the Care of Older Adults with Multimorbidity (2012). Patient-centered care for older adults with multiple chronic conditions: a stepwise approach from the American geriatrics society. J Am Geriatr Soc.

[6] World Health Organization. World report on ageing and health. 2015. http://www.who.int/ageing/events/world-report-2015-launch/en/. Accessed 10 May 2018

[7] Rechel B, Grundy E, Robine JM, Cylus J, Mackenbach JP, Knai C, McKee M (2013). Ageing in the European Union. Lancet.

[8] Michel JP, Huber P, Cruz-Jentoft AJ (2008). Europe-wide survey of teaching in geriatric medicine. J Am Geriatr Soc.

[9] Gordon AL, Blundell AG, Gladman JR, Masud T (2010). Are we teaching our students what they need to know about ageing? Results from the UK National Survey of Undergraduate Teaching in Ageing and Geriatric Medicine. Age Ageing.

[10] Singler K, Sieber CC, Biber R, Roller RE (2013). Considerations for the development of an undergraduate curriculum in geriatric medicine. Gerontology.

[11] Singler K, Holm EA, Jackson T, Robertson G, Müller-Eggenberger E, Roller RE (2015). European postgraduate training in geriatric medicine: data of a systematic international survey. Aging Clin Exp Res.

[12] Kredo T, Bernhardsson S, Machingaidze S, Young T, Louw Q, Ochodo E, Grimmer K (2016). Guide to clinical practice guidelines: the current state of play. Int J Qual Health Care.

[13] Wolfson D, Santa J, Slass L (2014). Engaging physicians and consumers in conversations about treatment overuse and waste: a short history of the choosing wisely campaign. Acad Med.

[14] American Board of Internal Medicine Foundation. Choosing Wisely. Facts and figures. 2018. http://www.choosingwisely.org/our-mission/facts-and-figures. Accessed 8 Apr 2018

[15] Levinson W, Born K, Wolfson D (2018). Choosing wisely campaigns: a work in progress. JAMA..

[16] European Union of Medical Specialists (UEMS) (2008) Geriatrics Section. Definition of geriatrics. http://uemsgeriatricmedicine.org/www/land/definition/english.asp. Accessed 25 Aug 2018

[17] American Board of Internal Medicine Foundation (2017) Choosing Wisely. Promoting conversations between patients and clinicians. http://www.choosingwisely.org. Accessed 8 Apr 2017

[18] Clinical Epidemiology Program of the Centro de Investigación Biomédica en Red de Epidemiología y Salud Pública (2017) DianaHealth—dissemination of initiatives to analyse appropriateness in Healthcare. http://dianasalud.com/index.php. Accessed 8 Apr 2017

[19] Otte J (2017) Less is more medicine: projects & initiatives. http://www.lessismoremedicine.com/projects/. Accessed 8 Apr 2017

[20] Arbeitsgemeinschaft der Wissenschaftlichen Medizinischen Fachgesellschaften (2017). http://www.awmf.org/leitlinien/awmf-regelwerk/ll-entwicklung/awmf-regelwerk-01-planung-und-organisation/po-stufenklassifikation/klassifikation-s3.html. Accessed 8 Apr 2017

[21] Österreichische Gesellschaft für Geriatrie und Gerontologie (2018). https://www.geriatrie-online.at/gesellschaft/vorstand/. Accessed 8 May 2018

[22] Hsu CC, Sandford BA (2007) The Delphi technique: making sense of consensus. Practical Assessment Research and Evaluation. http://pareonline.net/pdf/v12n10.pdf. Accessed 4 Apr 2018

[23] Preston CC, Colman AM (2000). Optimal number of response categories in rating scales: reliability, validity, discriminating power, and respondent preferences. Acta Physiol (Oxf).

[24] Vernon W (2009). The Delphi technique: a review. Int J Ther Rehabil.

[25] Gogol M, Siebenhofer A (2016). Choosing Wisely – Gegen Überversorgung im Gesundheitswesen – Aktivitäten aus Deutschland und Österreich am Beispiel der Geriatrie. Wien Med Wochenschr.

[26] American Geriatrics Society Choosing Wisely Workgroup (2013). American Geriatrics Society identifies five things that healthcare providers and patients should question. J Am Geriatr Soc.

[27] Arbeitsgemeinschaft der Wissenschaftlichen Medizinischen Fachgesellschaften e.V. (AWMF) (2016) Gemeinsam Klug Entscheiden. http://www.awmf.org/medizin-versorgung/gemeinsam-klug-entscheiden.html. Accessed 4 Apr 2018

[28] Malhotra A, Maughan D, Ansell J, Lehman R, Henderson A, Gray M, Stephenson T, Bailey S (2015). Choosing Wisely in the UK: the Academy of Medical Royal Colleges’ initiative to reduce the harms of too much medicine. BMJ.

[29] Levinson W, Kallewaard M, Bhatia RS, Wolfson D, Shortt S, Kerr EA (2015). “Choosing Wisely”: a growing international campaign. BMJ Qual Saf.

[30] Strech D, Follmann M, Klemperer D, Lelgemann M, Ollenschläger G, Raspe H, Nothacker M (2014). When Choosing Wisely meets clinical practice guidelines. Z Evid Fortbild Qual Gesundhwes.

[31] McMahon LF, Beyth RJ, Burger A, Chopra V, Feldstein D, Korenstein D, Subramanian U, Sussman J, Petty B, Tice J (2014). Enhancing patient-centered care: SGIM and choosing wisely. J Gen Intern Med.

[32] Schuur JD, Carney DP, Lyn ET, Raja AS, Michael JA, Ross NG, Venkatesh AK (2014). A top-five list for emergency medicine: a pilot project to improve the value of emergency care. JAMA Intern Med.

[33] American Board of Internal Medicine Foundation (2018) Recommendations of American Geriatrics Society. http://www.choosingwisely.org/clinicianlists/#keyword=geriatric&parentSociety=American_Geriatrics_Society. Accessed 12 May 2018

[34] Choosing Wisely Canada (2018) Geriatrics. Five things physicians and patients should question. https://choosingwiselycanada.org/geriatrics/. Accessed 12 May 2018

[35] Choosing Wisely Australia (2018) Australian and New Zealand Society for Geriatric Medicine: tests, treatments and procedures clinicians and consumers should question. http://www.choosingwisely.org.au/recommendations/anzsgm. Accessed 12 May 2018

[36] Rossi PD, Bilotta C, Consonni D, Nobili A, Damanti S, Marcucci M, Mannucci PM, Mari D, REPOSI Investigators (2016). Predictors of clinical events occurring during hospital stay among elderly patients admitted to medical wards in Italy. Eur J Intern Med.

[37] Palese A, Gonella S, Moreale R, Guarnier A, Barelli P, Zambiasi P, Allegrini E, Bazoli L, Casson P, Marin M, Padovan M, Picogna M, Taddia P, Salmaso D, Chiari P, Frison T, Marognolli O, Benaglio C, Canzan F, Ambrosi E, Saiani L, ESAMED Group (2016). Hospital-acquired functional decline in older patients cared for in acute medical wards and predictors: findings from a multicentre longitudinal study. Geriatr Nurs.

[38] Bhattacharyya O, Schull M, Shojania K, Stergiopoulos V, Naglie G, Webster F, Brandao R, Mohammed T, Christian J, Hawker G, Wilson L, Levinson W (2016). Building bridges to integrate care (BRIDGES): incubating health service innovation across the continuum of care for patients with multiple chronic conditions. Healthc Q.

[39] Briggs AM, Valentijn PP, Thiyagarajan JA, Araujo de Carvalho I (2018). Elements of integrated care approaches for older people: a review of reviews. BMJ Open.

[40] Zikmund-Fisher BJ, Kullgren JT, Fagerlin A, Klamerus ML, Bernstein SJ, Kerr EA (2017). Perceived barriers to implementing individual Choosing Wisely^®^ recommendations in two national surveys of primary care providers. J Gen Intern Med.

[41] Schwartz AL, Landon BE, Elshaug AG, Chernew ME, McWilliams JM (2014). Measuring low-value care in Medicare. JAMA Intern Med.

